# Cognitive Trajectories and Dementia Risk: A Comparison of Two Cognitive Reserve Measures

**DOI:** 10.3389/fnagi.2021.737736

**Published:** 2021-08-25

**Authors:** Federico Gallo, Grégoria Kalpouzos, Erika J. Laukka, Rui Wang, Chengxuan Qiu, Lars Bäckman, Anna Marseglia, Laura Fratiglioni, Serhiy Dekhtyar

**Affiliations:** ^1^Aging Research Center, Department of Neurobiology, Care Sciences and Society, Karolinska Institutet and Stockholm University, Stockholm, Sweden; ^2^Centre for Cognition and Decision Making, Institute for Cognitive Neuroscience, National Research University Higher School of Economics, Moscow, Russia; ^3^Centre for Neurolinguistics and Psycholinguistics, Vita-Salute San Raffaele University, Milan, Italy; ^4^Stockholm Gerontology Research Center, Stockholm, Sweden; ^5^The Swedish School of Sport and Health Sciences, GIH, Stockholm, Sweden; ^6^Department of Medicine and Wisconsin Alzheimer’s Disease Research Center, University of Wisconsin School of Medicine and Public Health, Madison, WI, United States; ^7^Division of Clinical Geriatrics, Department of Neurobiology, Care Sciences and Society, Karolinska Institutet, Stockholm, Sweden

**Keywords:** cognitive reserve, dementia, cognitive change, life course, *residual-based* cognitive reserve, population-based cohort, structural MRI

## Abstract

**Background and Objectives:**

Cognitive reserve (CR) is meant to account for the mismatch between brain damage and cognitive decline or dementia. Generally, CR has been operationalized using proxy variables indicating exposure to enriching activities (*activity-based* CR). An alternative approach defines CR as residual variance in cognition, not explained by the brain status (*residual-based* CR). The aim of this study is to compare *activity-based* and *residual-based* CR measures in their association with cognitive trajectories and dementia. Furthermore, we seek to examine if the two measures modify the impact of brain integrity on cognitive trajectories and if they predict dementia incidence independent of brain status.

**Methods:**

We used data on 430 older adults aged 60+ from the Swedish National Study on Aging and Care in Kungsholmen, followed for 12 years. *Residual-based* reserve was computed from a regression predicting episodic memory with a brain-integrity index incorporating six structural neuroimaging markers (white-matter hyperintensities volume, whole-brain gray matter volume, hippocampal volume, lateral ventricular volume, lacunes, and perivascular spaces), age, and sex. *Activity-based* reserve incorporated education, work complexity, social network, and leisure activities. Cognition was assessed with a composite of perceptual speed, semantic memory, letter-, and category fluency. Dementia was clinically diagnosed in accordance with DSM-IV criteria. Linear mixed models were used for cognitive change analyses. Interactions tested if reserve measures modified the association between brain-integrity and cognitive change. Cox proportional hazard models, adjusted for brain-integrity index, assessed dementia risk.

**Results:**

Both reserve measures were associated with cognitive trajectories [β × time (top tertile, ref.: bottom tertile) = 0.013; 95% CI: –0.126, –0.004 (*residual-based*) and 0.011; 95% CI: –0.001, 0.024, (*activity-based*)]. *Residual-based*, but not *activity-based* reserve mitigated the impact of brain integrity on cognitive decline [β (top tertile × time × brain integrity) = –0.021; 95% CI: –0.043, 0.001] and predicted 12-year dementia incidence, after accounting for the brain-integrity status [HR (top tertile) = 0.23; 95% CI: 0.09, 0.58].

**Interpretation:**

The operationalization of reserve based on residual cognitive performance may represent a more direct measure of CR than an *activity-based* approach. Ultimately, the two models of CR serve largely different aims. Accounting for brain integrity is essential in any model of reserve.

## Introduction

Aging is associated with gray- and white-matter lesions, atrophy, and functional disruptions that affect most areas of the brain (Walhovd et al., 2011). These changes have been linked to decline in several cognitive functions, as well as an increased risk of dementia (Frisoni et al., 2010; Kalpouzos and Nyberg, 2012; Lindenberger, 2014). However, a remarkable degree of inter-individual variability has been observed in the trajectories of cognitive decline and in the timing of dementia onset that cannot be accounted for by the brain parameters alone (Cosentino and Stern, 2019). Indeed, structural shrinkage, synaptic loss, and white matter degradation, are thought to be counteracted by the mechanisms that involve preservation, repair, or replenishment of neural resources (Cabeza et al., 2018).

A widely adopted model of resilience in cognitive aging and dementia (Nyberg et al., 2012) is that of *cognitive reserve* (Barulli and Stern, 2013; Stern et al., 2020) (CR). It assumes that some individuals are capable of coping with cognitive demands better than others in the face of brain-integrity loss (Stern, 2009). CR is presumed to act through two mechanisms: *neural reserve*, the efficiency or capacity of pre-existing functional brain networks; and *neural compensation*, the ability to use alternative cognitive strategies or neural pathways to circumvent deterioration (Steffener et al., 2011). Although the premise of CR has been generally accepted, operationalizing the construct has proven to be a challenge (Pettigrew and Soldan, 2019). The most commonly adopted approach to date has been to use prior stimulating experiences (most often education) as a proxy of reserve (Janse, 2012). Recently, this approach has been advanced in a life-course framework whereby, in addition to education, occupational complexity, social network, and engagement in leisure activities have been combined into a life-long indicator of CR (Wang et al., 2017; Dekhtyar et al., 2019). In line with this work, a scale for assessing cognitive reserve that incorporates many suspected contributors from different life stages has been developed (Nucci et al., 2012), validated (León et al., 2014), and adapted to several contexts (Maiovis et al., 2016; Altieri et al., 2018).

However, relying on recollected historical accounts of prior stimulating activities, which are at best an *indirect* proxy of reserve, may also introduce the risk of reverse causation and recall bias. Therefore, a more *direct* measure of CR that is also not dependent on self-reported information is highly warranted. An alternative approach has been suggested in which reserve is defined as the discrepancy between expected cognitive performance, given the level of brain integrity, and actual performance (Reed et al., 2010). Characterized as *residual* variance in cognitive performance, not explained by individual neuropathology and demographics, this operationalization has been suggested by some to offer a more precise measurement of reserve (Bocancea et al., 2021). The *residual-based* measure of CR, initially developed in a clinical sample of AD patients, has rarely been utilized in population-based aging cohorts. Importantly, it remains to be compared with a conventional operationalization of CR based on lifelong experiences, in its capacity to predict cognitive trajectories, as well as incident dementia.

In this study we aim to (1) compare *residual-based* and *activity-based* measures of CR in their association with cognitive trajectories, (2) assess the two measures of reserve in their ability to modulate the association between brain integrity and cognitive change trajectories, and (3) investigate if *residual-based* and *activity-based* CR are associated with dementia incidence after accounting for the levels of brain integrity.

## Materials and Methods

### Participants

Participants in this population-based cohort study were from the Swedish National Study on Aging and Care in Kungsholmen (SNAC-K), a community-based, longitudinal cohort study of adults aged 60+ years, living at home or in an institution in the Kungsholmen district of Stockholm (Lagergren et al., 2004). SNAC-K participants were randomly selected from 11 age cohorts (60, 66, 72, 78, 81, 84, 87, 90, 93, 96, and 99+ years). The younger age cohorts (60–72 years) were re-examined every 6 years, whereas the older cohorts (78+ years) were followed up every 3 years. At baseline (March 2001–August 2004), 3,363 of the 4,590 eligible individuals (73.3%) underwent examination. The SNAC-K magnetic resonance imaging (MRI) subsample (*n* = 555) included participants who were non-institutionalized and free from dementia and disability, recruited between September 2001 and October 2003 (Ferencz et al., 2013). Of the 555 participants, 125 were excluded due to incompleteness or suboptimal quality of MRI data (*n* = 43), presence of neurological or psychiatric diseases (*n* = 64), questionable dementia (*n* = 5), or missing cognition data (*n* = 13) at baseline, resulting in 430 subjects eligible for inclusion (see Supplementary Figure 1 for flowchart). In this study, we used follow-up data on cognition and dementia from four waves after the baseline assessment, resulting in a mean follow-up of 12 years. SNAC-K was approved by the Regional Ethical Review Board in Stockholm and written informed consent was obtained from participants or their next of kin.

### Cognitive Assessment

At baseline and at each follow-up wave, participants were administered a cognitive test battery according to a standardized procedure (Laukka et al., 2020). From the cognitive battery, five domains were available: *perceptual speed* [digit cancellation (Zazzo, 1974) and pattern comparison (Salthouse and Babcock, 1991)], *episodic memory* [word recall and word recognition (Laukka et al., 2013)], *semantic memory* (Dureman, 1960; Nilsson et al., 1997), *letter fluency* (A and F), and *category fluency* (animals and professions). For a more detailed description of the cognitive battery, see Laukka et al. (2013). In the analysis of cognitive trajectories, we used a composite index of cognitive performance computed as the average of *z*-scores for the domains of perceptual speed, semantic memory, letter fluency, and category fluency. A composite score of episodic memory was used in the operationalization of *residual-based* CR (see below).

In addition to a cognitive test battery, we also extracted information on Mini-Mental State Examination (MMSE), which was available at baseline and across all follow-up examinations. MMSE is a widely used, easy-to-administer, 30-item screening questionnaire, assessing various aspects of cognitive functioning, including temporal and spatial awareness, memory, language, and arithmetic (Folstein et al., 1975).

### Dementia Diagnosis

Dementia was clinically diagnosed according to DSM-IV criteria. A three-step procedure was employed, where two physicians working independently made a preliminary diagnosis and a third opinion was sought from the senior neurologist in the event of discordant assessments (Fratiglioni et al., 1997). For participants who died prior to follow-up assessment and did not receive a clinical diagnosis, dementia was ascertained through hospital records, hospital discharge registers, and death certificates.

### Neuroimaging Measures

Images were acquired with a Philips Intera 1.5T MRI scanner. The MRI protocol included an axial 3D T1-weighted fast-field-echo sequence (time of repetition (TR) 15 ms, time to echo (TE) 7 ms, flip angle (FA) 15°, field of view (FOV) 20, matrix 256 × 256), a fluid-attenuated inversion recovery (FLAIR) sequence (TR 6,000 ms, TE 100 ms, inversion time 1,900 ms, FA 90°, echo train length 21, FOV 184 × 230, matrix 204 × 256), and a proton density/T2-weighted fast-spin-echo sequence (TR 4,000 ms, TE 18/90 ms, FA 90°, echo train length 6, FOV 187.5 × 250, matrix 192 × 256, 5 mm slices, without the use of gap and angulation). Global white matter hyperintensities (WMH) volumes were manually drawn on FLAIR images and further interpolated on the corresponding T1-weighted images, to compensate for between-slices gap in FLAIR (intra-rater reliability assessed with Dice coefficient: 0.76, see Köhncke et al. (2016) for details). T1-weighted images were segmented into gray matter, white matter, and cerebrospinal fluid (CSF) using SPM12 in MATLAB R2012b (Statistical Parametric Mapping^1^), and subsequently visually inspected to check the quality of segmented images. Hippocampal volume (HCV) was extracted via an automated segmentation of the T1-weighted images (Fischl et al., 2002; Marseglia et al., 2019) using the Freesurfer 5.1 image analysis suite,^2^ and the lateral ventricular volume (LVV) was estimated via an automated segmentation performed with the ALVIN toolbox (Kempton et al., 2011). Number of lacunes, defined as round or ovoid fluid-filled cavities, 3–15 mm in diameter, consistent with a previous acute small deep brain infarct or hemorrhage in the territory of one perforating arteriole (Wardlaw et al., 2013), was assessed visually. The number and size of perivascular spaces (PVS) were also evaluated with a visual rating scale, and combined to derive a PVS score, as reported elsewhere (Laveskog et al., 2018). An index of brain integrity was computed using a structural equation model (SEM) by combining WMH volume, whole-brain gray matter volume (GMV), HCV, LVV, number of lacunes, and the PVS score (more estimation details in the secion “Statistical Analysis”).

### Cognitive Reserve Measures

#### *Residual-Based* Cognitive Reserve

*Residual-based* CR was defined as the discrepancy between *observed* and *predicted* levels of cognitive functioning, given the extent of observed brain integrity, and further accounting for age and sex. Consistent with previous work on *residual-based* CR (Reed et al., 2010; Zahodne et al., 2013), we used episodic memory as the index domain from which to derive reserve, as it is markedly impaired in aging. We fitted a linear regression model in which episodic memory performance was the dependent variable, whereas the observed level of brain integrity, based on the latent index derived from SEM (see section “Statistical Analysis”), was the independent variable; age and sex were included as covariates. From this linear model, we computed the residuals, the difference between observed and predicted levels of cognitive performance for each individual, which constituted our measure of *residual-based* CR.

#### *Activity-Based* Lifelong Cognitive Reserve

*Activity-based* CR incorporated four life experiences, hypothesized to contribute to the development of CR: Early-life education, midlife substantive work complexity, late-life leisure activities, and late-life social network. Information on life experiences was obtained from a nurse interview and accompanying questionnaires at the SNAC-K baseline assessment (Dekhtyar et al., 2019).

### Statistical Analysis

#### Deriving Cognitive Reserve Measures

We first used SEM to compute a latent brain-integrity index from six neuroimaging measures: WMH volume, whole-brain GMV, HCV, LVV, PVS score, and number of lacunes; age and sex were included as covariates. All volumetric measurements were corrected by the total intracranial volume (ICV) (Jack et al., 1989). Maximum likelihood with missing values (MLMV) estimation was used to estimate the model. Model fit was assessed using conventional criteria. Omitted paths were explored using modification indices and predicted values of the latent brain-integrity index were extracted. Next, we fitted a linear regression model with episodic memory score as the dependent variable and the latent brain-integrity index, sex, and age as independent variables. From this model, we calculated the residuals, which constituted the individual measure of *residual-based* CR. In the analyses, we used *residual-based* CR (mean: 0, range: –2.65, 2.23) both as continuous and categorical variable (tertile operationalization: low, moderate, and high reserve).

*Activity-based* CR measure was obtained using SEM that extracted a common latent factor from four stimulating life experiences: early life education, midlife substantive work complexity, late life leisure activities, and late life social network. A value of the latent variable was predicted for each individual, and the resulting continuous variable, *activity-based* CR, was approximately normally distributed with a mean of 0 (range: –3.28, 2.91). For the analyses, *activity-based* CR was considered continuously and categorically as tertiles.

#### Predicting Longitudinal Trajectories of Cognition

Next, we tested whether the two CR measures were associated with cognitive change over 12 years of follow-up. Cognitive trajectories were assessed using separate linear mixed-effects models with maximum-likelihood estimation, including the following factors as fixed effects: age, sex, follow-up time, brain-integrity index, CR indicator (*residual-based* or *activity-based*), alongside an interaction term for CR and time. Random effects for individual intercepts and slopes over time were also included. Predicted margins of cognitive trajectories were computed from the model using a tertile operationalization of CR.

#### Assessing CR as a Modulator of the Impact of Brain Integrity on Cognition

We investigated the role of the CR measures in modulating the relationship between the brain-integrity index and cognitive trajectories over time, by fitting two linear mixed-effects regression models (one for *residual-based* CR, the other for *activity-based* CR) with the composite cognitive score as the dependent variable. Independent variables included main effects of age, sex, follow-up time, brain-integrity index, and corresponding CR indicator, as well as the three-way interaction among brain-integrity index, the corresponding CR measure, and time.

#### Investigating Dementia Incidence in Relation to Two CR Measures

Cox proportional hazard models were used to assess the relative risk of dementia over 12 years in relation to the two CR measures. Separate models were estimated for *activity-based* and *residual-based* CR, and both included controls for age, sex, and brain-integrity index. Proportionality assumption was tested using Schoenfeld residuals. Follow-up time was computed as time since baseline until dementia diagnosis, death, or the last examination.

#### Sensitivity Analyses

In addition to cognitive test score trajectories, we also examined MMSE change over time. The reason for this analysis was to mitigate potential circularity bias, whereby *residual-based* CR was derived from a cognitive domain (episodic memory) and was subsequently related to a set of cognitive domains, which themselves may be correlated with the one used in the derivation of CR. Notably, the correlation between episodic memory at baseline and the composite score of four cognitive domains was only 0.35 when averaged over the follow-up (correlation between baseline episodic memory and mean MMSE over the follow-up was 0.31). Using MMSE also increased statistical power, as it had better coverage over the follow-up than cognitive assessment. In another sensitivity analysis, we derived *residual* CR using performance in all five cognitive domains, rather than just episodic memory, and related this index with MMSE change and dementia incidence over time. This ensured that a full spectrum of cognitive performance was considered both in the derivation of CR and in the outcome analysis, while circularity was mitigated by using different families of tests and outcomes.

#### Data Availability

Data are from the Swedish National Study on Aging and Care in Kungsholmen (SNAC-K^3^). Applications for data use can be submitted at https://www.snac-k.se/application/registration.php. For more information, contact Maria Wahlberg (Maria.Wahlberg@ki.se) at the Aging Research Center, Karolinska Institute.

## Results

Baseline characteristics of the study population are presented in Table 1. Individuals with higher scores on *residual-based CR* were on average more educated, had higher work complexity, larger social network, and more intact cognitive functioning (measured with MMSE).

**TABLE 1:** Baseline characteristics of the study population according to *residual-based* CR tertile.

Variables	*Residual-based* CR tertiles				
	
	Total sample (*N* = 430)	Tertile 1 (lowest CR)	Tertile 2 (medium CR)	Tertile 3 (highest CR)	Between-group comparison
	
	Mean (SD)	Mean (SD)	Mean (SD)	Mean (SD)	*p*-value
Age	70.43 (8.90)	70.36 (8.68)	70.81 (9.28)	70.13 (8.79)	0.99
Sex (proportion females)	58.6%	59.03%	57.34%	59.44%	0.93
MMSE (baseline)	29.14 (1.03)	28.86 (1.17)	29.06 (0.96)	29.51 (0.82)	0.000****
Education (years)	12.66 (4.31)	12.03 (4.09)	12.39 (4.28)	13.56 (4.43)	0.007***
Work complexity score (0–10)	5.13 (1.79)	4.87 (1.78)	5.13 (1.65)	5.39 (1.90)	0.049**
Leisure activities score (0–6)	2.75 (1.46)	2.68 (1.45)	2.78 (1.36)	2.78 (1.57)	0.807
Social network score (z-score)	0.15 (0.50)	0.04 (0.55)	0.20 (0.44)	0.21 (0.49)	0.005***
Perivascular spaces score	18.72 (5.09)	18.65 (5.02)	19.09 (5.21)	18.42 (5.04)	0.545
Total number of lacunes	0.29 (0.83)	0.27 (0.89)	0.33 (0.79)	0.28 (0.81)	0.805
Lateral ventricles volume (ICV-adjusted; in mL)	38.76 (16.89)	38.52 (17.92)	38.92 (17.09)	38.84 (15.70)	0.978
Hippocampal volume (ICV-adjusted; in mL)	7.53 (0.82)	7.55 (0.88)	7.50 (0.79)	7.54 (0.81)	0.884
Whole-brain gray-matter volume (ICV-adjusted; in mL)	551.81 (53.05)	552.56 (51.44)	552.06 (56.09)	550.81 (51.85)	0.96
White-matter hyperintensities volume (ICV-adjusted; in mL)	5.54 (9.15)	5.13 (9.28)	5.86 (8.87)	5.64 (9.34)	0.788

*Abbreviations: CR, cognitive reserve; SD, standard deviation; MMSE, mini-mental state examination; ICV, intracranial volume.*

^∗^*P* < 0.1; ^∗∗^*P* < 0.05; ^∗∗∗^*P* < 0.01; ^****^P < 0.001, two-tailed.

### Deriving Cognitive Reserve Measures

**FIGURE 1:**
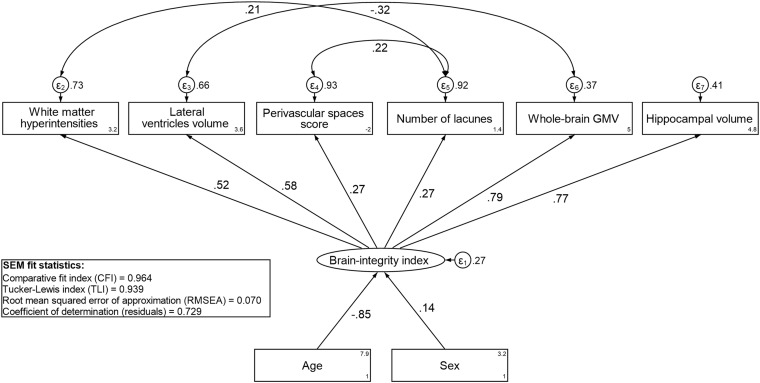
Standardized estimates from the best-fitting structural equation model (SEM) for the brain-integrity index.

To derive *residual-based* CR, we first constructed a latent index of brain integrity. The best-fitting SEM for the brain-integrity index is presented in Figure 1. The model fit the data well (CFI = 0.964; TLI = 0.939; RMSEA = 0.07). The factor loadings were highest for GMV and HCV, followed by WMH and LVV, and lowest for lacunes and PVS score. A value of the latent brain-integrity index was predicted for each individual. The resulting continuous variable was used as independent variable in the regression in which episodic memory was the dependent variable, and age and sex were included as covariates. The brain-integrity index was positively related to episodic memory performance (*p* < 0.01) and the residuals extracted from this model constituted the measure of *residual-based* CR. The correlation between the *residual-based* CR and the *activity-based* CR indicators was positive and statistically significant (*r* = 0.14; *p* = 0.004; see Supplementary Figure 2 for the SEM model used to derive *activity-based* CR).

### Predicting Longitudinal Trajectories of Cognition

**FIGURE 2:**
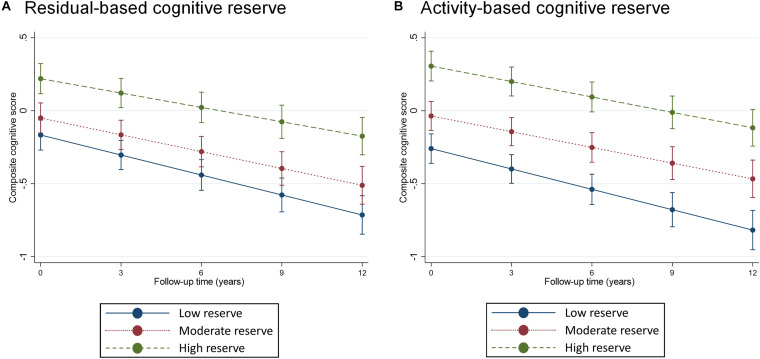
Predicted margins of cognitive change, measured using composite cognitive score, estimated separately for tertiles of *residual-based* (panel **A**) and activity-based (panel **B**) reserve. Predicted margins estimated from fully adjusted models presented in Table 2.

A continuous operationalization of *residual-based* CR did not yield an association with cognitive change trajectories (Table 2). Using a categorical operationalization of *residual-based* CR (tertile split) revealed that, relative to individuals in the lowest tertile of *residual-based* CR, those in the highest tertile exhibited slower cognitive decline over time (β for interaction with time: 0.013; *p* < 0.05). Predicted margins for cognitive trajectories according to tertiles of *residual-based* CR are presented in Figure 2A.

**TABLE 2:** Estimates from the linear mixed models predicting composite cognitive performance over time based on *residual*- and *activity-based* CR (continuous and in tertiles).

Variables	Subjects, N	Model estimates			
		
		*Residual-based* CR		*Activity-based* CR	
			
		β	[95% CI]	β	[95% CI]
CR (continuous)	430	0.222****	0.151, 0.292	0.205****	0.155, 0.254
CR (continuous) × time	430	0.005	–0.002, 0.011	0.005**	0.001, 0.009
**CR tertile**					
Lowest tertile	144	Referent			
Middle tertile	143	0.117	–0.028, 0.262	0.224***	0.082, 0.365
Highest tertile	143	0.386****	0.241, 0.532	0.566****	0.420, 0.711
**CR tertile × time**					
Lowest tertile × time	144	Referent			
Middle tertile × time	143	0.007	–0.005, 0.020	0.011	–0.003, 0.024
Highest tertile × time	143	0.013**	0.001, 0.025	0.011*	–0.001, 0.024

*Estimates come from separate linear mixed models.*

*The composite cognitive score was computed as the average of the *z*-scores of four cognitive domains: perceptual speed, semantic memory, category fluency, and letter fluency.*

*Episodic memory, used to derive the *residual-based* CR measure, was excluded from the composite cognitive score.*

*The models were adjusted for age, sex, time, and brain-integrity index (a latent factor incorporating six neuroimaging measures).*

*Abbreviations: CI, confidence interval; CR, cognitive reserve.*

^∗^*P* < 0.1; ^∗∗^*P* < 0.05; ^∗∗∗^*P* < 0.01; ^****^P < 0.001, two-tailed.

*Activity-based* CR exhibited a largely similar pattern of association with composite cognitive trajectories as did *residual-based* reserve. One difference was that, for *activity-based* CR, we also found a statistically significant association between a continuous operationalization and cognitive change (although the point estimate was identical to that of *residual-based* CR: β for interaction with time: 0.005, Table 2). Furthermore, relative to the bottom tertile, the top tertile of *activity-based* CR was only marginally associated with rate of cognitive decline (*p* = 0.08), although the point estimate (0.11) was quite similar to that of *residual-based* CR (0.13). Predicted margins for cognitive trajectories according to tertiles of *activity-based* CR are presented in Figure 2B.

### Examining CR as Modulator in the Brain Integrity-Cognitive Change Association

**FIGURE 3:**
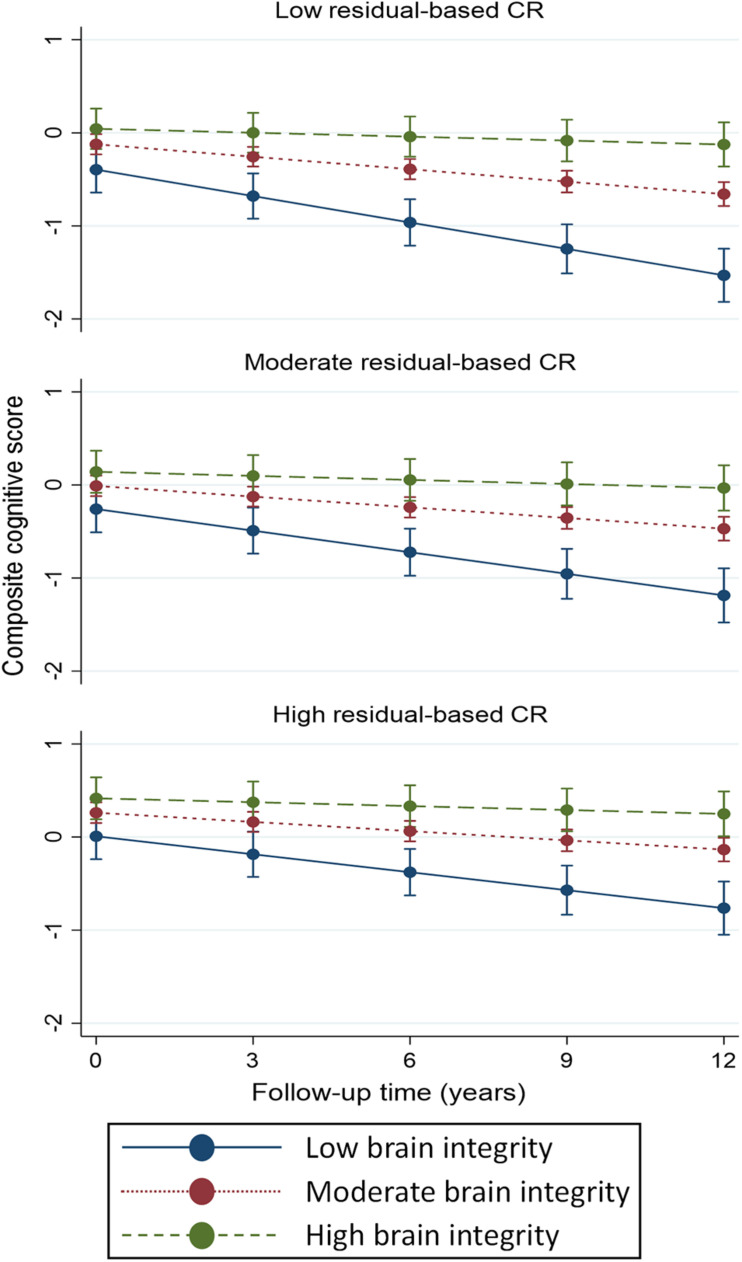
Predicted margins of cognitive change, based on composite cognitive score, in response to different levels of brain-integrity index, computed according to tertiles of *residual-based* CR. Predicted margins estimated from fully adjusted models presented in Table 3. Levels of brain-integrity index were defined as the 10th (low), 50th (moderate), and 90th (high) percentile.

Finally, we tested three-way interactions: CR (separately for *activity-* and *residual-based*) × brain-integrity index × time, to assess if CR modulated the impact of brain parameters on cognitive trajectories (Table 3). We found a statistically significant interaction among continuous *residual-based* CR and brain-integrity index over time (β = –0.011; *p* < 0.05), as well as a marginally statistically significant interaction among the top tertile of *residual-based* CR, brain-integrity index, and time (β = –0.021; *p* = 0.059; reference: low reserve × brain integrity × time). The margins plot of interaction (Figure 3) revealed that cognitive decline in response to impaired brain integrity was less pronounced in those with high *residual-based* CR than in those with low *residual-based CR.* In contrast, neither continuous nor categorical operationalizations of *activity-based* CR modulated the impact of brain integrity on composite cognitive trajectories.

**TABLE 3:** Estimates from linear mixed models investigating three-way interactions among CR [estimated separately for *residual*- and *activity-based* CR (continuous and in tertiles)], brain-integrity index, and time.

Variables	Model estimates			
	
	*Residual-based* CR		*Activity-based* CR	
		
	β	[95% CI]	β	[95% CI]
CR (continuous) × brain-integrity index × time	−0.011**	–0.022, –0.001	–0.001	–0.009, 0.007
**CR tertile × brain-integrity index × time**				
Lowest tertile × brain-integrity index × time	Referent			
Middle tertile × brain-integrity index × time	–0.012	–0.035, 0.01	0.004	–0.019, 0.027
Highest tertile × brain-integrity index × time	−0.021*	–0.043, 0.001	0.001	–0.023, 0.025

*Estimates come from separate linear mixed models.*

*Dependent variable: composite cognitive performance over 12 years computed as the average of the *z*-scores of four cognitive domains: perceptual speed, semantic memory, category fluency, and letter fluency.*

*Episodic memory, which was used to derive the *residual-based* CR measure, was excluded from the composite cognitive score to avoid circularity.*

*The models were adjusted for age, sex, time, and brain-integrity index (a latent factor incorporating six neuroimaging measures).*

*Abbreviations: CI, confidence interval; CR, cognitive reserve.*

^∗^*P* < 0.1; ^∗∗^*P* < 0.05; ^∗∗∗^*P* < 0.01; ^****^P < 0.001, two-tailed.

### Investigating Dementia Incidence in Relation to Two CR Measures

After a median of 11.5 years of follow-up (range: 0.76–15.7 years) of 419 participants (4218 person-years), 43 dementia cases were ascertained (incidence rate: 10.2 cases per 1000 person-years, 95% CI: 7.6–13.7). A continuous operationalization of *residual-based* CR was associated with a reduced hazard of dementia even after adjusting for the brain-integrity index (HR: 0.46; 95% CI: 0.33–0.66; Table 4). A model employing a tertile operationalization indicated that risk reduction was especially pronounced at the top third of *residual-based* CR (HR: 0.23; 95% CI: 0.09–0.58; reference: bottom tertile of *residual-based* CR, brain-integrity-adjusted model). The magnitude of risk reduction was less pronounced for *activity-based* CR and was no longer statistically significant upon further adjustment for the brain-integrity index. Proportionality assumption was not violated in any of the models.

**TABLE 4:** Hazard ratios for dementia incidence over 12 years according to *residual-* and *activity-based* CR (continuous and in tertiles).

Variables	Subjects, N	Cases, N	Model estimates			
			
					Additionally adjusted	
			Age and sex adjusted		for brain-integrity index	
				
			Hazard ratio	[95% CI]	Hazard ratio	[95% CI]
***Residual-based* CR**						
CR (continuous)	419	43	0.43****	0.3, 0.61	0.46****	0.33, 0.66
**CR tertile**						
Lowest tertile	138	24	Referent			
Middle tertile	139	13	0.52*	0.26, 1.03	0.57	0.29, 1.15
Highest tertile	142	6	0.22****	0.09, 0.54	0.23**	0.09, 0.58
***Activity-based* CR**						
CR (continuous)	419	43	0.76**	0.58, 0.99	0.81	0.62, 1.05
**CR tertile**						
Lowest tertile	141	21	Referent			
Middle tertile	139	15	0.72	0.37, 1.43	0.71	0.37, 1.39
Highest tertile	139	7	0.42*	0.17, 1.01	0.48	0.2, 1.16

*Cox PH models with age- and sex adjustment, as well as with additional adjustment for brain-integrity index (a latent factor incorporating six neuroimaging measures).*

*Abbreviations: CI, confidence interval; CR, cognitive reserve.*

^∗^*P* < 0.1; ^∗∗^*P* < 0.05; ^∗∗∗^*P* < 0.01; ^****^P < 0.001, two-tailed.

### Sensitivity Analyses

Replacing composite cognitive performance with MMSE did not affect principal findings. *Residual-based* CR was associated with a slower decline in MMSE score over the follow-up (both continuously and categorically: top tertile vs. bottom). A similar pattern was observed for *activity-based* CR, although the middle tertile of *activity-based* CR also exhibited an association with MMSE change, relative to the lowest tertile (Supplementary Table 1 and Supplementary Figure 3).

In the modulation analysis using MMSE as outcome (Supplementary Table 2 and Supplementary Figure 4), a three-way interaction *residual-based* CR × brain-integrity-index × time was statistically significant using continuous operationalization (*p* < 0.05), and marginally significant using categorical operationalization (*p* = 0.06 for the middle tertile and *p* = 0.09 for the top tertile). Consistent with the original findings, no such modulation was observed when considering *activity-based* CR (either continuous or categorical).

Re-estimating MMSE trajectories and dementia incidence using residual performance in all cognitive domains, rather than just episodic memory, did not alter the main findings. This alternative *composite-based residual* CR exhibited a statistically significant association with MMSE change in both continuous and categorical operationalizations (Supplementary Table 3 and Supplementary Figure 5). *Composite-based residual* CR measure also modified the impact of brain integrity on MMSE trajectories: a three-way interaction CR × brain-integrity-index × time (*p* < 0.05) emerged for both continuous and categorical operationalizations (Supplementary Table 4 and Supplementary Figure 6). Finally, consistent with the original findings, dementia risk was reduced in those with higher scores on *composite-based residual* CR, even in models adjusted for the brain-integrity index (Supplementary Table 5).

## Discussion

In this longitudinal population-based study of older adults, we found that a measure of cognitive reserve based on residual cognitive performance, unaccounted for brain integrity and sociodemographics, was associated with cognitive trajectories over a 12-year follow-up period. Furthermore, *residual-based* CR moderated the association between brain integrity and cognitive trajectories, such that in those with higher *residual-based* CR, the association between impaired brain integrity and cognitive decline was weakened compared to those with lower *residual-based* CR. Importantly, higher scores on *residual-based* CR were associated with 12-year hazard of dementia, even after accounting for brain-integrity levels. Conversely, although higher levels of *activity-based* CR were also associated with slower cognitive decline, this operationalization of CR neither modified the impact of brain integrity on the rate of cognitive change, nor was it associated with dementia occurrence net of the brain-integrity status.

*Residual-based* CR has been linked to cognitive trajectories in a handful of studies (Reed et al., 2010; Zahodne et al., 2013, 2015; Hohman et al., 2016; Habeck et al., 2017; Bettcher et al., 2019), and our findings are generally consistent with these earlier reports. The investigations with the most similar designs to the one reported here are Reed et al. (2010) and Zahodne et al. (2013). In a study of 305 adults varying in cognitive status, episodic-memory-based *residual* CR was linked to 3-year trajectories in executive function (Reed et al., 2010), whereas a study on 703 older adults, free from dementia at baseline, found differences in 3-year language ability trajectories according to *residual-based* CR (also derived using episodic memory) (Zahodne et al., 2013). Our study extends this literature in several important ways: (1) we examined cognitive trajectories over a prolonged follow-up period (12 years) in a population-based setting; (2) we were able to relate *residual-based* CR to a composite index of cognition based on measures of perceptual speed, verbal fluency, and semantic memory, as opposed to just executive function or language ability as used previously; (3) we extended the set of brain-integrity measures in deriving our *residual-based* measure of CR, incorporating WMH volume, whole-brain GMV, HCV, LVV, PVS, and lacunes; and (4) we examined 12-year dementia incidence in relation to *residual-based* CR. Incorporating MMSE trajectories and computing an alternative CR indicator based on residual performance in composite cognition, provided an important sensitivity test for the operationalization of *residual-based* CR adopted in this study.

A *residual-based* approach has been utilized in other studies, although they differ from ours in several important respects. One was based on cross-sectional data and derived *residual-based* CR from brain-integrity measures, without taking age into account (Habeck et al., 2017). Two other studies (Zahodne et al., 2015; Bettcher et al., 2019) examined changes in *residual-based* CR in relation to changes in cognition and brain atrophy. Finally, one study derived *residual-based* CR using cerebrospinal fluid markers of brain integrity (Hohman et al., 2016), although these findings were likely affected by circularity bias, as executive function and memory performance featured both in the derivation of CR measure and in the outcome analysis. Circularity might be a concern for our findings too, even though we excluded episodic memory from the outcome analysis. We aimed to further mitigate circularity through sensitivity analyses in which MMSE was used in place of cognition, as well as by analyzing dementia incidence. Notably, the correlation between episodic memory and composite cognitive performance over the follow-up was 0.35 (*r* = 0.31 for MMSE over time), suggesting that the bias due to circularity is unlikely to be considerable. Collectively, the present findings along with those from prior work discussed above highlight the utility of the *residual-based* approach in measuring cognitive reserve.

We made a further contribution by contrasting *residual-* and *activity-based* CR in the same study. Whereas both measures were associated with cognitive trajectories, only *residual-based* CR modified the brain integrity-cognitive change association and predicted dementia incidence after accounting for the brain-integrity levels. An apparent superior performance of *residual-based* CR is consistent with the findings of a recent meta-analysis that provided pooled estimates on MCI or dementia conversion across studies utilizing different reserve operationalizations (Nelson et al., 2021). The fact that *activity-based* CR did not yield modulatory or risk-reducing influences in our study could be due to the lack of precision in identifying reserve from diverse activities that may have differential contribution to the underlying construct. For instance, education or work complexity may affect cognitive outcomes through socioeconomic influences on health behaviors or access to material resources. These may in turn promote resistance to primary vascular pathologies, but ultimately play a lesser role in the resilience of cognitive function (Arenaza-Urquijo and Vemuri, 2018). Using a SEM-derived measure of *activity-based* reserve that focuses on common variance across all contributors while eliminating the variance that is unique to each one, has likely concealed their distinct influences. Specifically designed CR scales and questionnaires represent an alternative to SEM-based methods worthy of consideration in future studies (Nucci et al., 2012). We opted for a SEM-based measure here in order to (1) ensure comparability with our own and others’ prior work that also used SEM-based indexes of CR (Dekhtyar et al., 2019; Xu et al., 2019); (2) attenuate measurement error in observable factors contributing to CR, and (3) to integrate the impact of contributors not readily assessed in existing scales (e.g., social support).

Notably, the correlation between the two measures of reserve was weak (0.14), which compares to a correlation between *residual-based* CR and education of just 0.09 from a previous study (van Loenhoud et al., 2017). This suggests that the two CR measures likely incorporate distinct compensatory influences, and future studies ought to explore their unique neural bases. With respect to the seemingly limited relevance of *activity-based* reserve, the role of stimulating activities, particularly education, in age-related cognitive decline has been questioned in several studies (Berggren et al., 2018; Seblova et al., 2020). A recent comprehensive review concluded that cognitive stimulation likely relates to late-life cognition by affecting peak cognitive levels early in life, rather than the differential rates of decline during adulthood and aging (Lövdén et al., 2020). Although we documented an association between *activity-based* CR and cognitive trajectories and dementia in minimally adjusted models, the fact that it exhibited no mitigating effects against brain-integrity deterioration and lost its predictivity of dementia after brain integrity was accounted for, is in line with these recent appraisals of the role of stimulating activities.

Our findings provide input for the advancement of the CR theory, which continues to undergo considerable debate (Jones et al., 2011; Cabeza et al., 2018, 2019; Stern et al., 2019). On the one hand, we underscore the value of a *residual-based* approach which arguably offers a measure of reserve directly linked to its operational definition: the discrepancy between observed and expected cognitive performance, for a given level of brain integrity. However, *residual-based* CR can also be criticized for being dependent on the extent and quality of input parameters in the predictive model of cognition. In our study, it likely incorporated variance associated with unmeasured pathology that may affect episodic memory (notably: amyloid, tau, and TDP43), as well as the variance due to all other unobserved correlates of test performance. Thus, the association between this indicator and cognitive outcomes may not truly reflect reserve pathways, although it should be noted that a recent meta-analysis found *residual-based* CR to be associated with reduced progression to MCI or dementia, even in studies accounting for AD biomarkers (Nelson et al., 2021). Ultimately, *residual-* and *activity-based* operationalizations represent complementary approaches that can serve different aims. The former can be helpful for the prediction of future accelerated decline and dementia by identifying those with unexplained excessive cognitive deficits; information that can be especially useful for clinicians. The latter may give insight into how CR is formed in the first place, rather than providing its instantaneous measure. However, for either of these approaches to be consistent with the model of cognitive reserve, they must incorporate brain status in their operational definition; CR mechanisms should not be proposed when brain-integrity measures are not available at all, or are limited to a just a handful of markers that are insufficient to measure brain-integrity status (Habeck et al., 2017; Stern et al., 2020).

A strength of the present study is the longitudinal population-based design with long-term follow-up for cognition and dementia. The inclusion of a wide range of neuroimaging, cognitive, clinical, and life-experience measures is a further strength. Limitations include potential selectivity of healthier participants in the SNAC-K MRI subsample, which likely led to an underestimation of reported associations. Higher resolution MRI scans (as opposed to the 1.5T images used here) could have yielded a more detailed assessment of brain-integrity status, improving the specificity of our findings. By focusing on composite assessments, we strived for a holistic view of both brain integrity and cognition, which may have obscured important fine-grain detail, that ought to be explored further. For instance, the differential factor loadings of HCV (high), WMH (moderate), and PVS (low) to the brain-integrity index reported here, deserve attention in future studies looking to derive more specific measures of reserve. The absence of assessments of leisure participation and social network from before late life could also be a limitation. Finally, we used a more conventional regression-based technique to derive *residual-based* CR as opposed to SEM (Reed et al., 2010), and our residual estimate likely contain more measurement error. A linear regression approach, however, has been shown to be a reliable alternative to a latent-variable operationalization in a previous study (Zahodne et al., 2015).

## Conclusion

In conclusion, we showed that *residual-based* CR derived in a population-based study of older adults was (1) associated with cognitive trajectories over 12 years of follow-up, (2) mitigated the impact of impaired brain integrity on cognitive decline, and (3) predicted dementia incidence even after accounting for brain-integrity status. In contrast, an *activity-based* measure derived from stimulating life experiences neither mitigated the brain integrity-cognitive change association, nor did it emerge as a predictor of dementia independent of brain integrity. Our findings provide insight into future applications of CR models of cognitive change and dementia. Both approaches possess unique advantages that can be tailored to address different aims. Ultimately, any model of CR needs to consider brain integrity, and the term *reserve* should be used when the measure in question modifies the link between brain integrity and cognitive outcomes or predicts dementia independent of brain integrity. In our study, only a *residual-based* measure of reserve fulfilled this requirement.

## Data Availability Statement

The data analyzed in this study is subject to the following licenses/restrictions: Data are from the Swedish National Study on Aging and Care in Kungsholmen (SNAC-K; https://www.snac-k.se/). Data access is conditional on approval from the SNAC-K database committee. Applications for data use can be submitted at https://www.snac-k.se/application/registration.php. For more information, contact Maria Wahlberg (Maria.Wahlberg@ki.se) at the Aging Research Center, Karolinska Institute.

## Ethics Statement

The studies involving human participants were reviewed and approved by Regional Ethical Review Board in Stockholm. The patients/participants provided their written informed consent to participate in this study.

## Author Contributions

SD, FG, and LF: conception and design of the study. LF, GK, and EJL: acquisition of data. All authors: analysis or interpretation of data, drafting or revising the manuscript for intellectual content figures, contributed to the article, and approved the submitted version.

## Conflict of Interest

The authors declare that the research was conducted in the absence of any commercial or financial relationships that could be construed as a potential conflict of interest.

## Publisher’s Note

All claims expressed in this article are solely those of the authors and do not necessarily represent those of their affiliated organizations, or those of the publisher, the editors and the reviewers. Any product that may be evaluated in this article, or claim that may be made by its manufacturer, is not guaranteed or endorsed by the publisher.

## Footnotes

1
https://www.fil.ion.ucl.ac.uk/spm/

2
http://surfer.nmr.mgh.harvard.edu/

3
https://www.snac-k.se/
