# Supplementary material for: Cognitive Trajectories and Dementia Risk: A Comparison of Two Cognitive Reserve Measures
Source: Front Aging Neurosci. 2021 Aug 25;13:737736. doi: 10.3389/fnagi.2021.737736 (PMC8424183; doi:10.3389/fnagi.2021.737736)
Supplement: Supplementary file 1 [file Data_Sheet_1.docx]

**SUPPLEMENTARY MATERIALS**

**Table S1.** Estimates from the linear mixed models predicting MMSE performance over time based on residual- and activity-based CR (continuous and in tertiles)

| Variables | Subjects, N | Model estimates | | | | | |
| --- | --- | --- | --- | --- | --- | --- | --- |
|  |  | *Residual-based* CR | | | | *Activity-based* CR | |
|  |  | β | | [95% CI] | | β | [95% CI] |
| CR (continuous) | 430 | 0.367**** | | 0.236, 0.499 | | 0.108** | 0.011, 0.204 |
| CR (continuous) X time | 430 | 0.076**** | | 0.033, 0.119 | | 0.06**** | 0.03, 0.09 |
| CR tertile |  |  |  | |  |  |  |
| Lowest tertile | 144 | Referent | | | | | |
| Middle tertile | 143 | 0.19 | | -0.08, 0.459 | | 0.056 | -0.219, 0.332 |
| Highest tertile | 143 | 0.655**** | | 0.386, 0.924 | | 0.245* | -0.038, 0.528 |
| CR tertile X time |  |  | | |  |  |  |
| Lowest tertile X time | 144 | Referent | | | | | |
| Middle tertile X time | 143 | 0.067 | | -0.020, 0.155 | | 0.103** | 0.012, 0.193 |
| Highest tertile X time | 143 | 0.142**** | | 0.055, 0.229 | | 0.167**** | 0.078, 0.255 |

The models were adjusted for age, sex, time, and brain-integrity index (a latent factor incorporating six neuroimaging measures)
Abbreviations: CI, confidence interval; CR, cognitive reserve; MMSE, mini-mental state examination
*P < 0.1; **P < 0.05; ***P < 0.01; ****P < 0.001, two-tailed

**Table S2.** Estimates from the linear mixed models investigating three-way interactions between CR (estimated separately for *residual-* and *activity-based* CR [continuous and in tertiles]), brain-integrity index, and time for MMSE trajectories.

| Variables | Model estimates | | | | | |
| --- | --- | --- | --- | --- | --- | --- |
|  | *Residual-based* CR | | *Activity-based* CR | | | |
|  | β | [95% CI] | β | | [95% CI] | |
| CR (continuous) X brain-integrity index X time | -0.079** | -0.153, -0.006 | -0.032 | | -0.086, 0.023 | |
| CR tertile X brain-integrity index X time |  | | |  | |  |
| Lowest tertile X brain-integrity index X time | Referent | | | | | |
| Middle tertile X brain-integrity index X time | -0.147* | -0.303, 0.008 | -0.112 | | -0.268, 0.045 | |
| Highest tertile X brain-integrity index X time | -0.133* | -0.285, 0.019 | -0.033 | | -0.199, 0.134 | |

The models were adjusted for age, sex, time and brain-integrity index (a latent factor incorporating 6 different neuroimaging measures)
Abbreviations: CI, confidence interval; CR, cognitive reserve; MMSE, mini-mental state examination
*P < 0.1; **P < 0.05; ***P < 0.01; ****P < 0.001, two-tailed

**Table S3.** Estimates from the linear mixed models predicting MMSE performance over time based on composite-based residual CR (continuous and in tertiles)

| Variables | Subjects, N | Model estimates | | | |
| --- | --- | --- | --- | --- | --- |
|  |  | *Composite-based* *residual* CR | | | |
|  |  | β | | | [95% CI] |
| CR (continuous) | 430 | 0.713**** | | | 0.525, 0.900 |
| CR (continuous) X time | 430 | 0.111**** | | | 0.051, 0.172 |
| CR tertile |  |  |  |  |  |
| Lowest tertile | 144 | Referent | | | |
| Middle tertile | 143 | 0.592**** | | | 0.325, 0.86 |
| Highest tertile | 143 | 0.956**** | | | 0.691, 1.221 |
| CR tertile X time |  |  | | |  |
| Lowest tertile X time | 144 | Referent | | | |
| Middle tertile X time | 143 | 0.109** | | | 0.021, 0.197 |
| Highest tertile X time | 143 | 0.113*** | | | 0.027, 0.199 |

The models were adjusted for age, sex, time, and brain-integrity index (a latent factor incorporating six neuroimaging measures)
Abbreviations: CI, confidence interval; CR, cognitive reserve; MMSE, mini-mental state examination
*P < 0.1; **P < 0.05; ***P < 0.01; ****P < 0.001, two-tailed

**Table S4.** Estimates from the linear mixed models investigating three-way interactions between *composite-based residual* CR (continuous and in tertiles), brain-integrity index, and time for MMSE trajectories.

| Variables | Subjects, N | Model estimates | | |
| --- | --- | --- | --- | --- |
|  |  | *Composite-based* *residual* CR | | |
|  |  | β | | [95% CI] |
| CR (continuous) X brain-integrity index X time | 430 | -0.131** | | -0.236, -0.025 |
| CR tertile X brain-integrity index X time |  |  |  |  |
| Lowest tertile X brain-integrity index X time | 144 | Referent | | |
| Middle tertile X brain-integrity index X time | 143 | -0.188** | | -0.347, -0.03 |
| Highest tertile X brain-integrity index X time | 143 | -0.193** | | -0.342, -0.044 |

The models were adjusted for age, sex, time and brain-integrity index (a latent factor incorporating 6 different neuroimaging measures)
Abbreviations: CI, confidence interval; CR, cognitive reserve; MMSE, mini-mental state examination
*P < 0.1; **P < 0.05; ***P < 0.01; ****P < 0.001

**Table S5:** Dementia incidence over 12 years in the SNAC-K MRI subsample according to *composite-based residual* CR (continuous and in tertiles)

| Variables | Subjects, N | Cases, N | Model estimates | | | | | |
| --- | --- | --- | --- | --- | --- | --- | --- | --- |
|  |  |  | *Composite-based residual* CR | | | | | |
|  |  |  | Age and sex  adjusted | | | Additionally adjusted for brain-integrity index | | |
|  |  |  | Hazard Ratio | [95% CI] | | Hazard Ratio | | [95% CI] |
| CR (continuous) | 419 | 43 | 0.43*** | 0.26, 0.71 | | 0.43*** | | 0.26, 0.69 |
| CR tertile |  |  |  | |  | |  |  |
| Lowest tertile | 139 | 24 | Referent | | | | | |
| Middle tertile | 140 | 6 | 0.28*** | 0.11, 0.69 | | 0.26*** | | 0.1, 0.64 |
| Highest tertile | 140 | 13 | 0.44** | 0.22, 0.86 | | 0.39*** | | 0.2, 0.77 |

Cox PH models with basic adjustment, as well as additional adjustment for brain-integrity index (a latent factor incorporating six neuroimaging measures)
Abbreviations: CI, confidence interval; CR, cognitive reserve

*P < 0.1; **P < 0.05; ***P < 0.01; ****P < 0.001

**
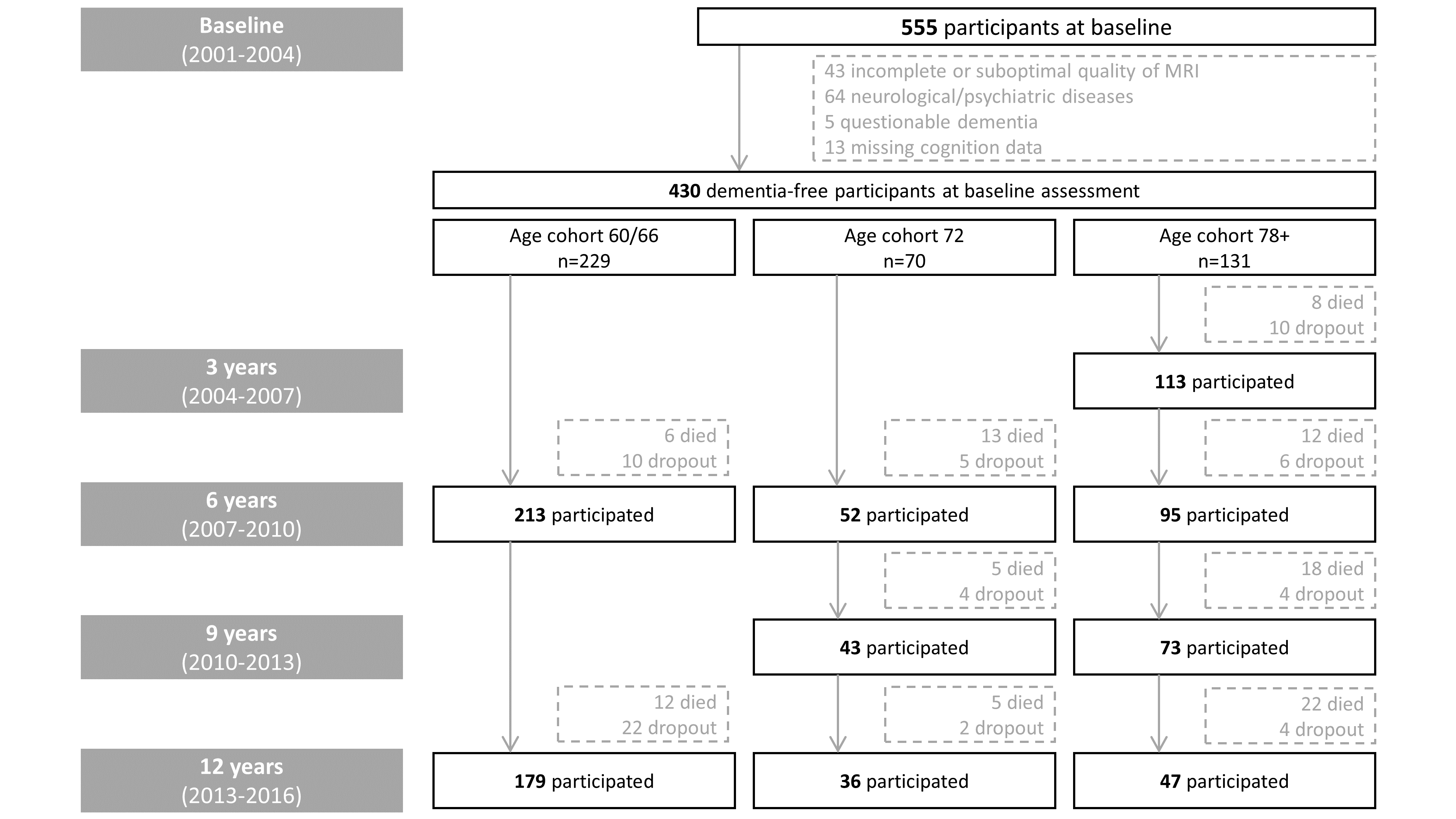
Figure S1.** Flowchart of study population in the SNAC-K MRI subsample.

**Figure S2.** Standardized estimates from the best-fitting structural equation model (SEM) for the *activity-based* cognitive reserve measure.

As the model only had one degree of freedom, the values of CFI, TLI, and RMSEA have little interpretational value. Model fit was subsequently assessed using a chi-squared statistic, which confirmed a satisfactory fit (χ^2^ = 0.22, df = 1, p = 0.641).
**Figure S3.** Predicted margins of cognitive change, measured using MMSE, estimated separately for tertiles of residual-based (panel A) and activity-based (panel B) reserve.

**Figure S4.** Predicted margins of cognitive change, based on MMSE, in response to different levels of brain-integrity index, computed according to tertiles of residual-based CR.

Levels of brain-integrity index were defined as the 10th (low), 50th (moderate) and 90th (high) percentile.

**Figure S5.** Predicted margins of cognitive change, measured using MMSE, estimated according to tertiles of composite-based residual CR.

Composite-based residual cognitive reserve was computed from the linear regression in which a composite score of perceptual speed, semantic memory, category fluency and letter fluency and episodic memory was the dependent variable, while brain-integrity index, age, and sex were the independent variables.

**Figure S6.** Predicted margins of cognitive change, based on MMSE, in response to different levels of brain-integrity index, computed according to tertiles of *composite-based residual* CR.

Composite-based residual cognitive reserve was computed from the linear regression in which a composite score of perceptual speed, semantic memory, category fluency and letter fluency and episodic memory was the dependent variable, while brain-integrity index, age, and sex were the independent variables.
Levels of brain-integrity index were defined as the 10th (low), 50th (moderate) and 90th (high) percentile.
